# Changes in Expression of Syndecans and Heparan Sulfate Biosynthesis Enzymes in Short-Term Streptozotocin-Induced Diabetic Rat Kidneys

**DOI:** 10.3390/cells15141277

**Published:** 2026-07-16

**Authors:** Tanja Čujić, Anita Racetin, Natalija Filipović, Sandra Kostić, Nela Kelam, Petar Todorović, Katarina Vukojević

**Affiliations:** 1 Department of Pulmonology, University Hospital of Split, 21000 Split, Croatia; tcujic@kbsplit.hr; 2 Department of Anatomy, Histology and Embryology, University of Split School of Medicine, 21000 Split, Croatia; anita.racetin@mefst.hr (A.R.); natalija.filipovic@mefst.hr (N.F.); sandra.kostic@mefst.hr (S.K.); nela.kelam@mefst.hr (N.K.); petar.todorovic@mefst.hr (P.T.); 3 Mediterranean Institute for Life Sciences, University of Split, 21000 Split, Croatia; 4 Center for Translational Research in Biomedicine, University of Split School of Medicine, 21000 Split, Croatia

**Keywords:** syndecan family members, heparan sulfate biosynthesis enzymes, diabetes, kidneys, rats

## Abstract

**Highlights:**

- Diabetes abolishes the normal maturational rise of renal SDC1 in rats.
- SDC2 remains inappropriately elevated in diabetic kidneys at two months.
- NDST1 is biphasic: raised at two weeks, then profoundly suppressed by two months.
- Public transcriptomes reproduce the syndecan changes and point to post-transcriptional NDST control.

**Abstract:**

Background: The aim of this study was to determine the temporal expression patterns of syndecan family members (SDC1, SDC2, SDC4) and heparan sulfate biosynthesis enzymes (NDST1, NDST2) in kidneys of diabetic rats and age-matched controls. Methods: Male Sprague–Dawley rats received intraperitoneal streptozotocin (55 mg/kg; DM1 group) or citrate buffer (control group). Kidney samples were harvested after 2 weeks and 2 months and processed for immunofluorescence. Results: SDC1 showed significant temporal upregulation in controls that was abolished in diabetic animals. SDC2 exhibited high early expression in the control group with significant decline as the kidneys matured but remained elevated in diabetic kidneys at 2 months compared to controls. SDC4 showed no significant difference between groups, though an age-related decrease was observed in controls. NDST1 was significantly upregulated in diabetic rats at 2 weeks, followed by profound suppression at 2 months (*p* < 0.0001). NDST2 showed modest but significant early elevation in diabetic animals. Transcript-level analysis of two independent public datasets of streptozotocin-induced diabetic rat renal cortex reproduced the principal directional findings—an early increase in *SDC1* and a progressive elevation of *SDC2*—while indicating post-transcriptional regulation of *SDC4* and the early *NDST* response. Conclusions: Diabetes disrupts normal temporal expression of syndecans and heparan sulfate biosynthesis enzymes in rat kidneys. Early compensatory upregulation of NDST1 and NDST2, followed by progressive NDST1 suppression, suggests a deteriorating heparan sulfate biosynthetic capacity, potentially contributing to the progression of diabetic nephropathy.

## 1. Introduction

Diabetes mellitus (DM) presents a significant global burden, with 537 million adults affected worldwide and projections reaching 784 million by 2045 [1]. Hyperglycemia drives increased inflammatory activity, endothelial dysfunction, and microvascular complications, contributing to diabetic nephropathy (DN), a well-defined complication characterized by glomerular hyperfiltration, basement membrane thickening, podocyte injury, mesangial expansion, and fibrosis [2,3,4]. Although the absolute burden of diabetic nephropathy is greater in type 2 diabetes owing to its far higher prevalence, the streptozotocin-induced type 1 model isolates the effect of chronic hyperglycemia itself, without the confounding obesity, insulin resistance, and dyslipidemia inherent to type 2 models. It therefore remains a standard, well-characterized system for dissecting the early extracellular-matrix remodeling that precedes overt nephropathy, which is the focus of the present study.

Syndecans are a family of four cell surface heparan sulfate proteoglycans (HSPGs) and major components of the endothelial glycocalyx [5,6]. SDC1 is expressed in epithelial linings [7], SDC2 on mesenchymal cells [8], SDC3 in neuronal tissue [9,10], and SDC4 ubiquitously [11,12]. Through their heparan sulfate (HS) glycosaminoglycan side chains, syndecans act as ECM receptors and growth factor co-receptors [13,14]. Hyperglycemia increases syndecan shedding, contributing to diabetic complications [15]. SDC1 serum levels correlate with microalbuminuria in both DM1 and DM2 [16,17,18], while SDC4 shedding has been implicated in glomerular filtration barrier dysfunction in early DN [19]. HS biosynthesis is regulated by N-deacetylase/N-sulfotransferases (NDSTs), with NDST1 and NDST2 being the predominant isoforms in adult tissues [20]. NDST activity is considered rate-limiting for HS sulfation. In diabetic conditions, hepatic NDST1 activity is suppressed by over 50% within two weeks of DM1 induction in rats [21], while endothelial-specific NDST1 ablation reduced glomerular inflammation and fibrosis in a murine DN model [22].

Despite growing evidence of syndecan and NDST involvement in diabetic complications, data on their temporal expression patterns specifically in the diabetic kidney remain scarce. In particular, the dynamic interplay between individual syndecan family members and NDST isoforms during the early and established phases of diabetic nephropathy has not been systematically characterized.

The present study was designed to investigate the temporal expression of SDC1, SDC2, SDC4, NDST1, and NDST2 in the kidneys of streptozotocin-induced diabetic rats and age-matched controls at 2 weeks and 2 months post-induction.

## 2. Materials and Methods

### 2.1. Ethics

This study was done following the guidelines of the Declaration of Helsinki. The Ethics Committee of the University of Split School of Medicine approved the experimental protocol of this study (protocol number: 2181-198-03-04-19-0061, class: 003-08/19-03/0003).

### 2.2. Experimental Animals

The Animal Facility of the University of Split School of Medicine provided the experimental animals used in this study. Male Sprague–Dawley rats were reared under controlled conditions, including a temperature of 22 ± 1 °C and a light program consisting of 12 h of light and 12 h of dark. The weights of the rats used in the study were between 160 and 180 g. A Type 1 diabetes mellitus (DM1) rat model was used for this experiment. For the induction, rats received an injection of 55 mg/kg streptozotocin freshly dissolved in citrate buffer into the peritoneal cavity, pH value of 4.5, after an overnight fast. Intraperitoneal injection of citrate buffer was used for the control (CTRL) group. The food used for feeding animals was a regular laboratory diet ad libitum (4RF21 GLP, Mucedola, Settimo Milanese, Italy), made of 9% fat, 27% proteins, and 64% carbohydrates. Plasma glucose and body mass index were closely measured in order to validate diabetes in animals. A single-touch glucometer (OneTouchVita, LifeScan, High Wycombe, UK) was used for measuring blood glucose levels taken from a single drop of blood collected from the tail vein. Animals with glucose levels above 16.5 mmol/L were considered diabetic and were subsequently included in further experiments. During the whole experiment, glucose levels were between 16.5 and 30 mmol/L. All of the rats in the experimental group developed symptoms of diabetes (high glucose concentration, polydipsia, and polyuria). For measuring their body weight, a weighing scale was used. The non-fasting plasma glucose levels at two weeks were 27 ± 3 mmol/L, and at two months 27 ± 2 mmol/L. The weight of diabetic rats at the start of the experiment was 160 ± 2 g, after two weeks 208 ± 9 g, and after two months 244 ± 7 g. The non-fasting plasma glucose levels of the rats in the control group were 7.5 ± 0.5 mmol/L at two weeks and 7.2 ± 0.4 mmol/L at two months. The weight of these rats was 167 ± 15 g at the beginning of the experiment, 250 ± 6 g after two weeks, and 466 ± 13 g after two months. Diabetic rats were allocated into two groups based on the duration of diabetes from the injection of STZ to the end of the experiment: two weeks (DM1 2 w) and two months (DM1 2 m). For each diabetic group, there was a matched control group: two weeks (CTRL 2 w) and two months (CTRL 2 m). There were five animals in each group except the two months’ control group, which had seven animals.

### 2.3. Tissue Collection and Immunofluorescence

Rats were anesthetized using isoflurane (Forane, Abbott Laboratories, Queenborough, UK). Subsequently, rats were perfused with 300 mL of Zamboni’s fixative 4% paraformaldehyde and 15% picric acid in 0.1 M phosphate-buffered saline (PBS) followed by washing with 300 mL of saline at a pH of 7.4. Kidney samples were taken for further analysis and post-fixed in the same fixative. Afterwards, the tissue was embedded in paraffin blocks, which were cut into sections 5 μm thick. Following deparaffinization in xylol, the sections were rehydrated using ethanol and water, and cooked at 95 °C for 30 min in a water steamer in 0.01 M citrate buffer (pH 6.0). After cooling down to room temperature, the sections were washed in 0.1 M PBS, and to inhibit nonspecific staining, a protein-blocking solution (ab64226, Abcam, Cambridge, UK) was applied for 20 min. Following application of primary antibodies and an overnight incubation in a humidity chamber, materials were rinsed with PBS and incubated with secondary antibodies for one hour (Table 1). Sections were then rinsed with PBS and then stained with 4′,6-Diamidino-2-phenylindole (DAPI) in order to visualize nuclei. Slides were rinsed in PBS, covered by mounting media (Immuno-Mount, Thermo Shandon, Pittsburgh, PA, USA) and a coverslip. For the preadsorption test, each primary antibody was diluted in a blocking solution at the predetermined concentration. An appropriate peptide antigen was added to the solution with which sections were treated. There was no detected evidence of antibody staining, nonspecific binding of secondary antibodies, or false-positive results when primary antibodies were excluded from the immunofluorescence protocol.

### 2.4. Data Acquisition

The cortex of control and diabetic kidneys was examined and photographed using an Olympus BX51 epifluorescence microscope (Olympus, Tokyo, Japan) equipped with a Nikon DS-Ri2 digital camera (Nikon Corporation, Tokyo, Japan). Images were acquired using NIS-Elements F software version 4.30.01 (Nikon Instruments Inc., Tokyo, Japan) at a resolution of 1360 × 1024 pixels with identical exposure settings for all samples. SDC1, SDC2, SDC4, NDST1, and NDST2 were examined in 10 non-overlapping fields of the healthy control kidney samples and of diabetic kidneys at two different time points, two weeks and two months. Positive staining was observed as punctate or diffuse green or red staining.

### 2.5. Image Analysis of Area Percentage

To quantify the immunofluorescence signal of the researched proteins, we used ImageJ software version 1.54g (National Institutes of Health, Bethesda, MD, USA) to analyze the microphotographs. All images were processed in the same order. To minimize the fluorescence leakage, we removed the red countersignal from the green fluorescence and the green countersignal from the red fluorescence, after which we applied a median filter with a radius of 5.0 pixels. To identify the positive signal, we subtracted the non-filtered photos from the filtered images and then converted the resulting photos into 8-bit images. The triangle thresholding algorithm was used for sequential modification of each image. By using the “analyze particles” feature, we determined the percentage of fluorescence.

### 2.6. Statistical Analysis

GraphPad Prism version 9.0.0 software (GraphPad Software, San Diego, CA, USA) was used for statistical analysis. The results were expressed as the calculated percentage’s mean ± standard deviation. The Shapiro–Wilk test was used to assess the data’s distribution normality. A probability level (p) of less than 0.05 was used for characterization of each dataset pertaining to area percentage analysis, which was considered statistically significant. SDC1, SDC2, SDC4, NDST1, and NDST2 expression levels in the cortex of the kidneys of a healthy control group and a group with DM1 were analyzed and compared using a two-way analysis of variance (ANOVA) test followed by Sidak’s multiple comparison test. GraphPad Prism 9.0.0 was used for creating all graphs. Adobe Photoshop (Adobe, San Jose, CA, USA) was used for assembling plates. For presentation purposes, microphotographs were processed for background subtraction and contrast.

### 2.7. Rationale and Dataset Selection

To complement the immunofluorescence (IF) protein data with an independent assessment at the transcript level, publicly available gene-expression datasets were retrieved from the NCBI Gene Expression Omnibus (GEO; https://www.ncbi.nlm.nih.gov/geo/; accessed on 12 June 2026). Datasets were considered eligible when they fulfilled all of the following criteria: (i) species *Rattus norvegicus*; (ii) streptozotocin (STZ)-induced diabetes; (iii) renal cortical tissue (matching the compartment sampled in the present IF study); and (iv) availability of a control (non-diabetic/sham) group and a diabetic group with at least three biological replicates each. Two datasets met all criteria and represented two complementary disease phases: GSE131221 (early phase) and GSE7253 (mid phase). Both employ the same strain background (Sprague–Dawley) and STZ induction used in the present study, differing only in the exact post-induction interval and the transcriptomic platform (Table S1).

The mid-phase dataset (GSE7253; DataSet record GDS4038; Table S2) profiled the renal cortex of male Sprague–Dawley rats 6 weeks after streptozotocin (STZ)-induced diabetes on the Affymetrix Rat Genome 230 2.0 array, in a balanced 2 × 2 design crossing disease state (diabetic vs. non-diabetic) with age at onset (juvenile vs. adult; *n* = 3 per cell, 12 samples) [23]. The early-phase dataset (GSE131221; Table S3) profiled the renal cortex of male Sprague–Dawley rats on an Agilent 8 × 60 K microarray approximately 3 weeks after STZ induction, comprising a sham (*n* = 5) and a diabetic (*n* = 7) group; a pharmacologically treated arm present in the series was excluded, and only sham (control) and diabetic samples were compared [24].

The five proteins studied by immunofluorescence were queried using their rat gene symbols (*SDC1*, *SDC2*, *SDC4*, *NDST1*, *NDST2*) (Tables S2 and S3). To assess the wider pathway, the chain-polymerizing exostosins (*Ext1*, *Ext2*), the glucuronyl C5-epimerase (*GLCE*), the remaining N-deacetylase/N-sulfotransferases (*NDST3*, *NDST4*), and the O-sulfotransferases (*HS2ST1*, *HS3ST1*, *HS6ST1*), together with the neuronal syndecan *SDC3*, were additionally queried (Tables S4 and S5, Figure S1). *NDST3* and *NDST4* were not represented on either platform; *SDC3* was represented only on the mid-phase platform. Where multiple probes mapped to one gene, the probe with the highest mean signal was used as representative.

Normalized values were taken from each dataset as provided (signal intensity for GSE7253, log_2_ expression for GSE131221) without additional renormalization. Fold change is reported as diabetic relative to control. Because of the small group sizes, the primary comparison used the two-sided Mann–Whitney U test, complemented by Welch’s *t*-test; for the balanced GSE7253 design, a two-way ANOVA (disease, age, interaction) was additionally fitted on log_2_ values. Within each dataset, *p*-values were corrected for multiple comparisons using the Benjamini–Hochberg false-discovery-rate procedure, applied separately to the five core genes and to the extended panel. Analyses were performed in Python 3 (NumPy, SciPy, statsmodels); significance was set at *p* < 0.05.

## 3. Results

This study analyzed the immunofluorescence expression of SDC1, SDC2, SDC4 and the heparan sulfate biosynthesis enzymes NDST1 and NDST2 in the kidneys between the control and diabetic groups during periods of two weeks and two months’ post-induction of DM1. Results are expressed as a percentage of positively stained area.

**Figure 1:**
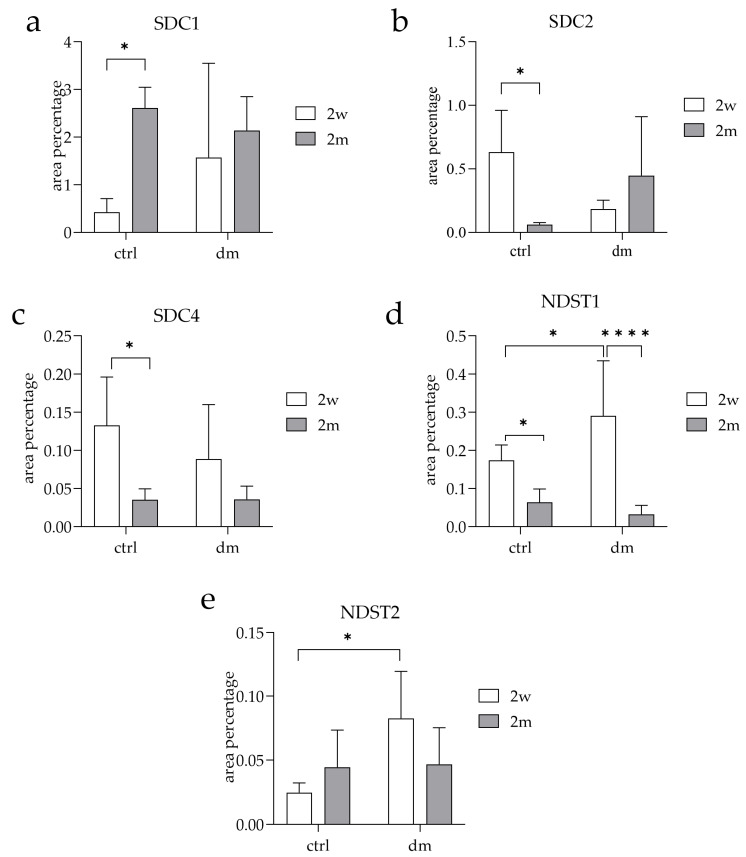
Area percentage of positive immunofluorescence signal for SDC1 (**a**), SDC2 (**b**), SDC4 (**c**), NDST1 (**d**), and NDST2 (**e**) in the kidney cortex of control (CTRL) and streptozotocin-induced diabetic (DM1) rats at two weeks (2 w) and two months (2 m). Ten representative, non-overlapping fields of cortex were analyzed per sample. Values are mean ± SD. Two-way ANOVA with Sidak’s post hoc test; * *p* < 0.05, **** *p* < 0.0001.

In our results, SDC1 showed a statistically significant increase in expression in the control group from 2 weeks to 2 months (Figure 1a, *p* < 0.05).

**Figure 2:**
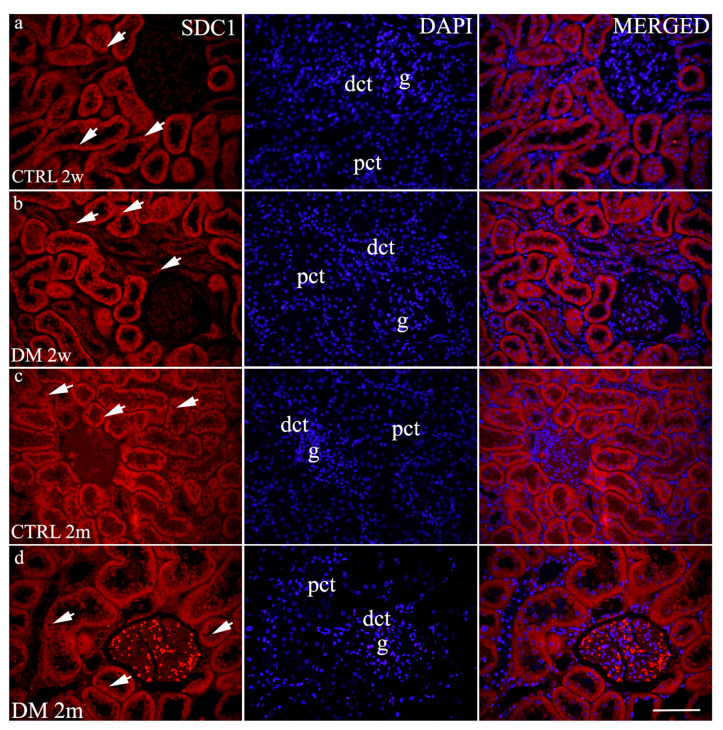
Immunofluorescence staining of Syndecan-1 (SDC1) in rat kidney cortex. Representative microphotographs of SDC1 immunofluorescence with DAPI nuclear counterstain in kidney cortex sections from control (CTRL; (**a**,**c**)) and streptozotocin-induced diabetic (DM1; (**b**,**d**)) rats at two weeks (2 w; (**a**,**b**)) and two months (2 m; (**c**,**d**)). White arrows indicate SDC1 signal in the epithelium of proximal (pct) and distal (dct) convoluted tubules. SDC1 signal is predominantly localized in tubular epithelial cells. Scale bar = 100 μm. g—glomerulus, pct—proximal convoluted tubule, dct—distal convoluted tubule.

However, in the DM1 group, the increase is blunted. Comparing both age groups, the expression of SDC1 was higher in the DM1 2 w group than in the age-matched controls, but lower in the DM1 2 m group compared to the age-matched control group, suggesting an early reactive response followed by progressive epithelial HSPG depletion (Figure 1a). Immunofluorescence images showed a similar expression pattern among all groups, with predominant localization in the epithelial cells of both proximal (pct) and distal (dct) tubules, while the glomerulus (g) showed mostly weak staining (Figure 2).

**Figure 3:**
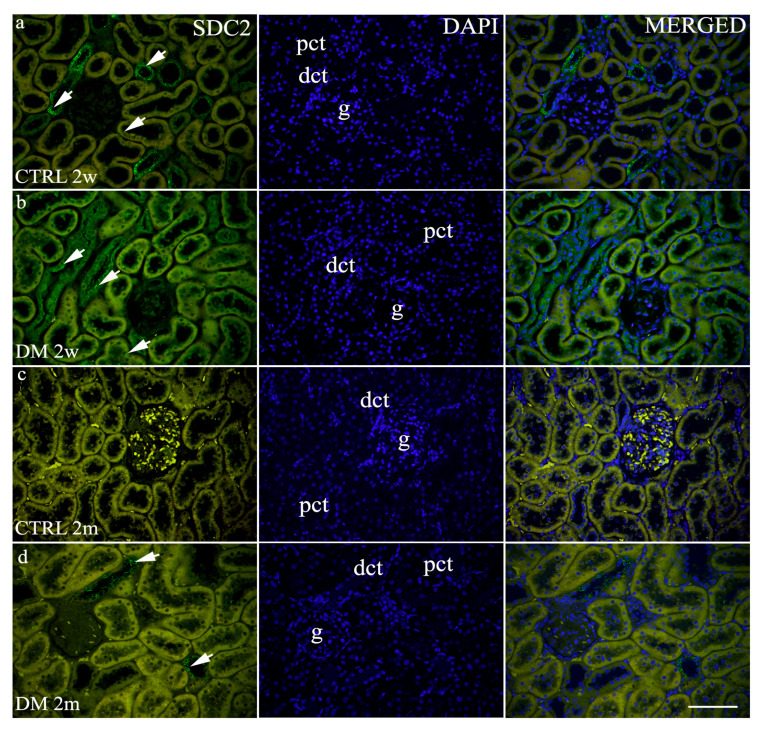
Immunofluorescence staining of Syndecan-2 (SDC2) in rat kidney cortex. Representative microphotographs of SDC2 immunofluorescence with DAPI nuclear counterstain in kidney cortex sections from control (CTRL; (**a**,**c**)) and streptozotocin-induced diabetic (DM1; (**b**,**d**)) rats at two weeks (2 w; (**a**,**b**)) and two months (2 m; (**c**,**d**)). SDC2 signal (arrows) is predominantly localized in the distal convoluted tubules. Scale bar = 100 μm. g—glomerulus, pct—proximal convoluted tubule, dct—distal convoluted tubule.

SDC2 exhibited high early expression in the control group, with significant decline as the kidneys matured from 2 weeks to 2 months (Figure 1b, *p* < 0.05). In contrast, kidneys of diabetic rats failed to downregulate SDC2, maintaining higher expression at 2 months than age-matched controls and showing a non-significant trend toward further increase compared to the DM1 2-week group. The declined expression of SDC2 in the DM1 2 w group compared to the age-matched control is noteworthy, although this was not a statistically significant finding. This sustained SDC2 elevation in the diabetic group might suggest the progression of interstitial fibrosis. SDC2 immunofluorescence displayed a markedly distinct distribution pattern from SDC1. In CTRL 2 w sections, the signal was most intense of all four groups, concentrated mostly in the distal convoluted tubules (arrows, Figure 3). This high early signal declined sharply by CTRL 2 m, where the cortex appeared almost with no staining. In the DM1 2 w group, the signal was weaker than in CTRL 2 w, but with the same appearance, mostly in the dct. In DM1 2 m sections, the SDC2 signal persisted in the dct, with arrows marking zones of retained staining that were absent in the corresponding CTRL 2 m preparations, visually confirming the failure to downregulate SDC2 and supporting a pattern consistent with progressive interstitial fibrosis (Figure 3).

**Figure 4:**
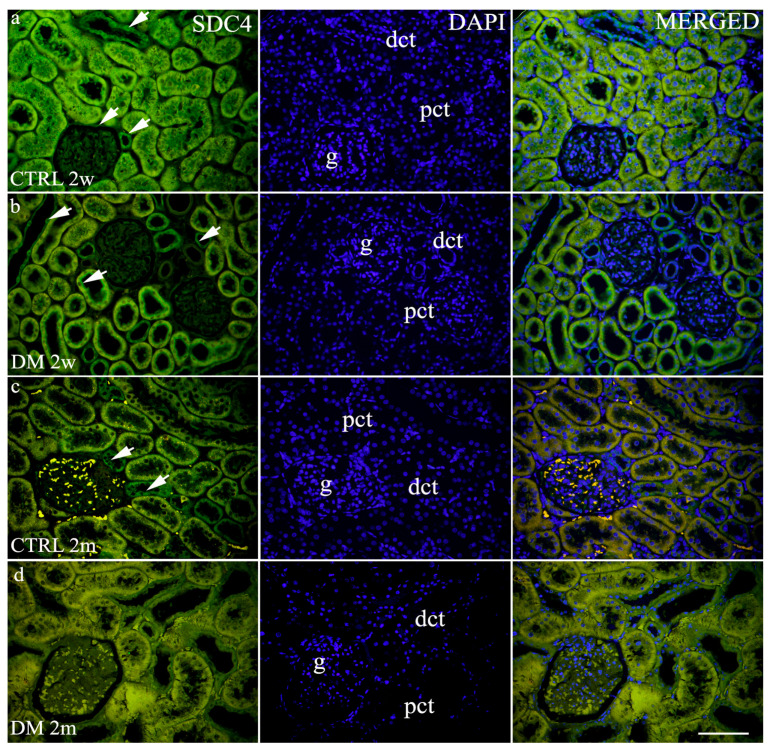
Immunofluorescence staining of Syndecan-4 (SDC4) in rat kidney cortex. Representative microphotographs of SDC4 immunofluorescence with DAPI nuclear counterstain in kidney cortex sections from control (CTRL; (**a**,**c**)) and streptozotocin-induced diabetic (DM1; (**b**,**d**)) rats at two weeks (2 w; (**a**,**b**)) and two months (2 m; (**c**,**d**)). SDC4 signal (arrows) is diffusely distributed across tubular compartments. Scale bar = 100 μm. g—glomerulus, pct—proximal convoluted tubule, dct—distal convoluted tubule.

No significant difference was found in the SDC4 kidney expression between the control and diabetic group at either time point, although SDC4 expression was slightly lower in diabetic animals at the 2 w time point than in the control group at 2 w. A statistically significant age-related decline in the expression of SDC4 was observed in the control group (Figure 1c, *p* < 0.05), with a parallel but non-significant trend in DM1 animals, suggesting that SDC4 kidney expression level is relatively preserved in DN, consistent with post-transcriptional maintenance of SDC4 protein, since transcript levels were modestly reduced rather than elevated. SDC4 immunofluorescence showed a more diffuse distribution than SDC2, covering tubular membranes and, to a variable extent, the Bowman capsule (Figure 4). In CTRL 2 w sections, the signal was moderate and relatively homogeneous across the cortex, with arrows marking the dct and parietal epithelial cells of the Bowman capsule. The DM1 2 w signal appeared slightly weaker. Notably, in CTRL 2 m groups, staining was accumulated mostly in the cells of dct but also on the apical membranes of pct. In DM1 2 m sections, tubular staining was diffusely faint (Figure 4).

**Figure 5:**
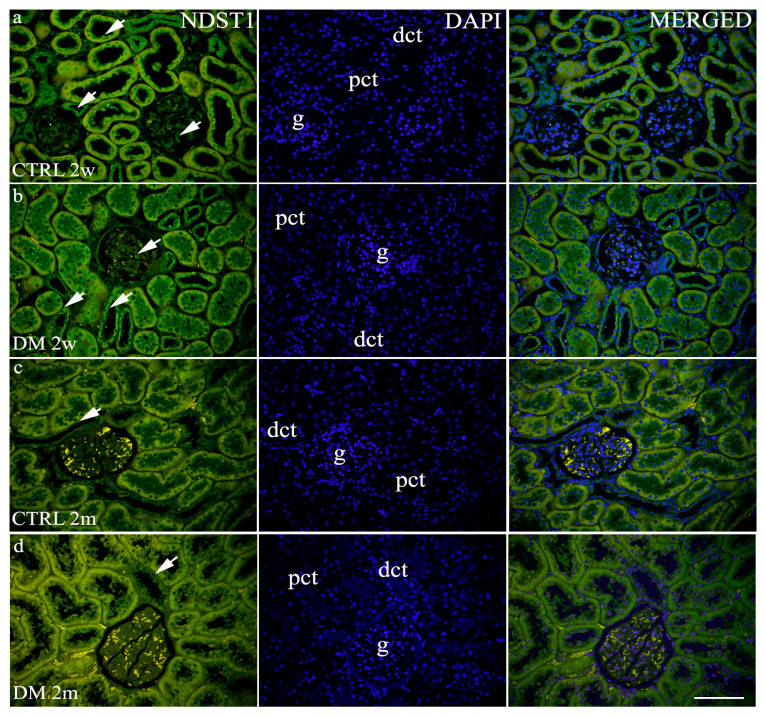
Immunofluorescence staining of N-deacetylase/N-sulfotransferase 1 (NDST1) in rat kidney cortex. Representative microphotographs of NDST1 immunofluorescence with DAPI nuclear counterstain in kidney cortex sections from control (CTRL; (**a**,**c**)) and streptozotocin-induced diabetic (DM1; (**b**,**d**)) rats at two weeks (2 w; (**a**,**b**)) and two months (2 m; (**c**,**d**)). NDST1 signal (arrows) is predominantly localized to tubular and occasionally glomerular cells. Scale bar = 100 μm. g—glomerulus, pct—proximal convoluted tubule, dct—distal convoluted tubule.

An interesting pattern was noticed in the percentage of positively stained area of NDST1. A significant developmental decline from 2 weeks to 2 months was observed in controls (*p* < 0.05). At 2 weeks, DM1 animals showed significantly elevated NDST1 compared to controls, indicative of a compensatory biosynthetic response (*p* < 0.05). However, by 2 months post-induction, DM1 expression of NDST1 was profoundly suppressed compared to the early DM1 group (*p* < 0.0001), pointing to exhaustion of this compensatory mechanism and a failure of HS N-sulfation capacity in established DN. The most striking expression dynamics were observed for NDST1. The signal was localized predominantly to tubular and occasionally glomerular cells (Figure 5). In CTRL 2 w sections, staining was predominant along the tubular membranes, and in the glomeruli. In DM1 2 w sections, the signal was visibly enhanced, the tubular cortex appeared more intensely stained (Figure 5b). In CTRL 2 m sections and DM1 2 m, overall signal dramatically declined (Figure 5c,d).

**Figure 6:**
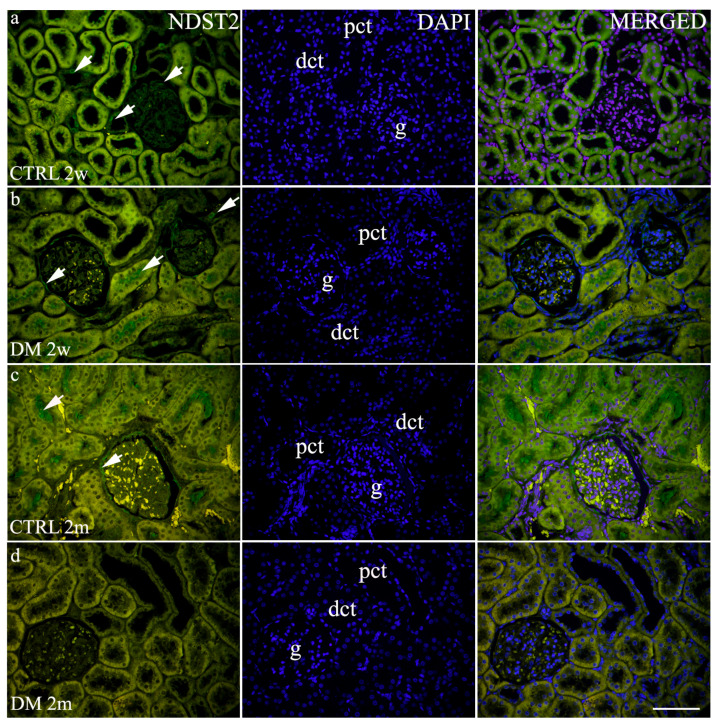
Immunofluorescence staining of N-deacetylase/N-sulfotransferase 2 (NDST2) in rat kidney cortex. Representative microphotographs of NDST2 immunofluorescence with DAPI nuclear counterstain in kidney cortex sections from control (CTRL; (**a**,**c**)) and streptozotocin-induced diabetic (DM1; (**b**,**d**)) rats at two weeks (2 w; (**a**,**b**)) and two months (2 m; (**c**,**d**)). NDST2 signal (arrows) is uniformly low across all groups. Scale bar = 100 μm. g—glomerulus, pct—proximal convoluted tubule, dct—distal convoluted tubule.

NDST2 expression was uniformly low across all groups. A modest but statistically significant higher expression was observed in DM1 animals at 2 weeks compared to controls (*p* < 0.05), mirroring the early NDST1 upregulation and suggesting a coordinated initial biosynthetic response. By 2 months, NDST2 levels were comparable between diabetic animals and age-matched controls. NDST2 immunofluorescence was uniformly low in intensity across all groups (Figure 6). In CTRL 2 w and DM1 2 w sections, the signal was faint and dispersed along tubular membranes (Figure 6a,b). In CTRL 2 m preparations, a prominent staining was observed on the apical membranes of pct and in the Bowman capsule. In DM1 2 m sections, almost no staining was observed (Figure 6d).

### In Silico Validation of the Immunofluorescence Findings

**Figure 7:**
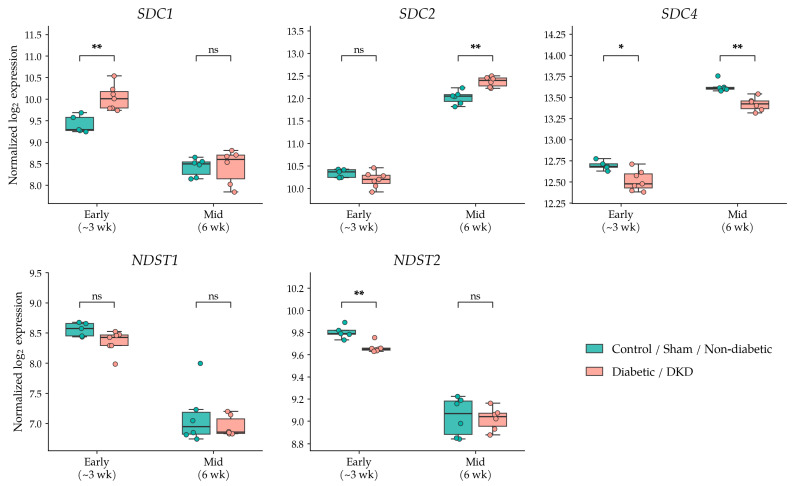
Transcript expression of the five studied genes (*SDC1*, *SDC2*, *SDC4*, *NDST1*, *NDST2*) in STZ-induced diabetic rat kidney cortex at the early phase (GSE131221, ~3 weeks; Sham vs. Diabetic) and mid phase (GSE7253, 6 weeks; Control vs. Diabetic). Boxplots show normalized log_2_ expression of the representative probe per gene; individual samples are overlaid, and mid-phase signal intensities are shown on a log_2_ scale. Significance (Mann–Whitney): * *p* < 0.05, ** *p* < 0.01, ns = not significant.

To assess whether the protein-level changes were mirrored at the transcript level, the five studied genes were examined in two independent public datasets of STZ-induced diabetic rat renal cortex (Figure 7; Supplementary Tables S2 and S3).

In the early-phase cortex, the *SDC1* transcript was markedly higher in diabetic than in the control cortex (1.53-fold; Mann–Whitney *p* = 0.003), the strongest signal in either dataset, and returned to control levels by the mid phase—reproducing the early reactive increase in SDC1 protein. Conversely, the *SDC2* transcript was unchanged early but significantly higher at the mid phase (1.27-fold; *p* = 0.004), matching the sustained increase in SDC2 protein in the diabetic cortex. The *SDC4* transcript was modestly but significantly lower at both phases (0.88- and 0.87-fold; *p* = 0.018 and *p* = 0.002), whereas SDC4 protein was preserved, indicating post-transcriptional maintenance rather than transcriptional upregulation. Finally, the early rise in NDST1 and NDST2 protein was not accompanied by a corresponding transcript increase (*NDST2* was in fact slightly lower early, 0.91-fold, *p* = 0.005), consistent with a predominantly post-transcriptional regulation of the early NDST response. The concordance between protein and transcript findings is summarized in Table 2.

## 4. Discussion

The present study examined the temporal expression of SDC1, SDC2, SDC4, NDST1, and NDST2 in rat kidneys at 2 weeks and 2 months following streptozotocin-induced diabetes, revealing distinct and divergent expression trajectories that reflect a progressive remodeling of the renal heparan sulfate proteoglycan landscape.

SDC1 showed a progressive increase in control kidneys from 2 weeks to 2 months, consistent with its role in epithelial integrity [7]. In diabetic animals, this upregulation was blunted, and at 2 months, SDC1 was lower than in age-matched controls. These findings align with clinical evidence that serum SDC1 is elevated in microalbuminuric type 1 diabetic patients [16] and independently predicts albuminuria in type 2 diabetes [18]. The early transient increase in SDC1 at 2 weeks may reflect an initial reactive response to hyperglycemic stress. Heparanase, upregulated by albumin and advanced glycation end-products in proximal tubular cells, further regulates SDC1 gene expression [25], providing a mechanistic link between metabolic injury and SDC1 loss.

In controls, SDC2 expression was high at 2 weeks and declined significantly by 2 months, consistent with its developmental regulation on mesenchymal cells [8]. In diabetic kidneys, this downregulation failed to occur, and SDC2 remained elevated at 2 months, with a persistent interstitial and peritubular immunofluorescence signal. Notably, Ramnath et al. reported downregulation of SDC2 mRNA in isolated diabetic glomeruli [19], suggesting compartment-specific regulation; the sustained elevation we observe in whole cortex likely predominantly reflects tubulointerstitial changes. SDC2 directly promotes TGF-β binding and matrix deposition in renal fibroblasts [26] and is overexpressed in fibrotic conditions [25], and its de novo induction in glomeruli as a compensatory response to SDC4 loss in nephrectomized syndecan-4-deficient mice paradoxically amplified pro-sclerotic TGF-β1 activity [27]. Together, these findings suggest that failure to downregulate SDC2 in our diabetic animals reflects a shift toward a fibrosis-permissive renal environment.

SDC4 showed no significant difference between diabetic and control kidneys at either time point, though controls displayed an age-related decline and a focal glomerular SDC4 accumulation at 2 months that was absent in diabetic animals. SDC4 is the predominant HSPG in the glomerular endothelial glycocalyx, and its MMP-mediated shedding contributes to albuminuria in early diabetic kidney disease [19]. Importantly, hyperglycemia per se does not drive SDC4 shedding from glomerular endothelial cells—IL-1β, via MMP9, is the primary stimulus [28], which may explain why total cortical SDC4 appears preserved despite established diabetes. An additional regulatory layer involves SDC1–SDC4 interdependence: SDC1 suppresses SDC4 expression through ERK1/2 and p38 MAPK pathways [29], so the SDC1 decline observed at 2 months of diabetes may partly maintain SDC4 levels through disinhibition. Furthermore, NDST1-mediated N-sulfation is critical for proper SDC4 localization and podocyte cell-matrix interactions [29], suggesting that the profound NDST1 suppression found in diabetic animals at 2 months may impair SDC4 function even where protein levels appear preserved.

NDST1 showed biphasic expression in diabetic kidneys: significant upregulation at 2 weeks, followed by profound suppression at 2 months. This early upregulation appears to be a kidney-specific compensatory response, contrasting with the hepatic NDST suppression (>50%) already evident at 2 weeks of STZ-induced diabetes in rats, where angiotensin II contributes to post-transcriptional inhibition [21]. A parallel early inductive response has been described in vascular injury, where NDST1 mRNA increased 20-fold in mouse vessels at 7–14 days post-injury [30]. The profound NDST1 suppression at 2 months has major functional consequences. NDST1 initiates N-sulfation reactions that generate ligand-binding sites on HS chains, and its loss produces under-sulfated, biologically less active HS [22]. Paradoxically, Talsma et al. demonstrated that endothelial-specific NDST1 ablation in murine STZ-induced DN completely prevented glomerular macrophage influx, complement deposition, glomerulosclerosis, and tubulointerstitial fibrosis [22], suggesting that properly sulfated endothelial HS may scaffold pro-inflammatory mediators. Whether the NDST1 suppression in our model represents a similarly ambiguous consequence, impairing barrier integrity while attenuating inflammatory recruitment, warrants further investigation. At the podocyte level, NDST1 deletion disrupts SDC4 clustering and focal adhesion signaling, impairing podocyte attachment and migration [31], adding another mechanism by which progressive NDST1 loss may compromise glomerular filtration barrier integrity in established DN.

NDST2 was uniformly low across all groups but showed a modest early elevation in diabetic animals at 2 weeks, paralleling NDST1 and suggesting a coordinated initial biosynthetic response. By 2 months, NDST2 levels equalized between groups. This is consistent with evidence that NDST2 contributes substantially to total NDST activity but does not independently affect HS structure as long as NDST1 is present; only in NDST1-deficient conditions does NDST2 become responsible for N-sulfation of residual low-sulfated HS [32]. The normalization of NDST2 at 2 months against a background of profound NDST1 suppression therefore implies failure of this compensatory mechanism, leaving renal HS N-sulfation capacity severely compromised in established diabetic nephropathy. In skin fibroblasts from diabetic patients, high glucose reduced NDST2 mRNA while NDST1 was unaffected [33], highlighting tissue-specific regulatory differences and underscoring the kidney-intrinsic nature of the early compensatory response we describe.

To place these findings in the context of the wider HS biosynthetic pathway, we additionally examined the chain-polymerizing exostosins (*EXT1*, *EXT2*), the glucuronyl C5-epimerase (*GLCE*), and the O-sulfotransferases (*HS2ST1*, *HS3ST1*, *HS6ST1*) at the transcript level in the same public datasets. The broader machinery was largely stable, with two modest but significant exceptions confined to the early phase: a small increase in *GLCE* and a decrease in *HS3ST1*, accompanied by a non-significant upward trend in *HS2ST1* and *HS6ST1*. These observations suggest that early diabetic HS remodeling is not strictly confined to the *NDST* isoforms and may extend to the O-sulfotransferase layer, although the changes are small and the syndecans remained the dominant and most consistent transcriptional signal. As transcript-level findings from independent cohorts, they are hypothesis-generating and warrant targeted confirmation in future work.

Several limitations should be acknowledged. First, immunofluorescence quantification of the whole kidney cortex limits compartment-specific resolution; future studies using laser-capture microdissection or cell-type-specific isolation would allow glomerular and tubulointerstitial contributions to be assessed separately. Second, the streptozotocin model represents type 1 diabetes and does not fully recapitulate the metabolic context of type 2 diabetic nephropathy. Third, orthogonal protein- and RNA-based confirmation on the same cohort was not possible: the tissue was perfusion-fixed with Zamboni’s fixative to preserve morphology for immunofluorescence, which precludes reliable Western blotting and quantitative PCR, and no unfixed or frozen tissue was available. To mitigate this, antibody specificity was verified by preadsorption controls, and the protein findings were validated at the transcript level using two independent public datasets; nonetheless, the immunofluorescence signal remains semi-quantitative, and the transcriptomic data should be regarded as supportive rather than confirmatory. Fourth, changes in *NDST* expression do not necessarily translate into altered heparan sulfate abundance or sulfation patterns; direct confirmation would require disaccharide compositional analysis or sulfation-specific anti-HS antibodies, and *NDST3* and *NDST4* were not represented on either transcriptomic platform. Finally, renal functional parameters (albuminuria, serum creatinine) were not measured in this cohort, so the functional implications of the observed changes are inferred from the literature rather than directly demonstrated.

## 5. Conclusions

This study shows that short-term streptozotocin-induced diabetes disrupts the normal temporal expression program of syndecans and heparan sulfate N-sulfotransferases in the rat renal cortex, in an isoform- and time-dependent manner. The maturational increase in SDC1 observed in control kidneys was abolished in diabetes, SDC2 remained inappropriately elevated at two months, and SDC4 protein was preserved despite a reduced transcript. Most strikingly, NDST1 followed a biphasic course—an early, compensatory rise at two weeks followed by profound suppression at two months—while NDST2 was only modestly and transiently elevated.

Independent transcript-level analysis of two public datasets reproduced the principal syndecan findings and indicated that the early NDST response and the maintenance of SDC4 are predominantly post-transcriptional. Taken together, these temporal changes suggest an initial compensatory effort to sustain heparan sulfate sulfation that ultimately fails, pointing to a progressive decline in heparan sulfate biosynthetic capacity that may contribute to the progression of diabetic nephropathy. Direct structural confirmation of altered heparan sulfate sulfation, together with correlation to renal functional endpoints, represents an important next step.

## Figures and Tables

**Table 1:** Primary and secondary antibodies used in the study.

	Antibodies	Host	Code No.	Dilution	Source
Primary	anti-SDC1	Mouse	ab34164	1:100	Abcam, Cambridge, UK
	anti-SDC2	Rabbit	ab191062	1:200	Abcam, Cambridge, UK
	anti-SDC4	Rabbit	ab24511	1:100	Abcam, Cambridge, UK
	anti-NDST1	Rabbit	ab129248	1:50	Abcam, Cambridge, UK
	anti-NDST2	Rabbit	ab151141	1:100	Abcam, Cambridge, UK
Secondary	Anti-Mouse IgG H&L (TRITC)	Goat	ab6786	1:400	Abcam, Cambridge, UK
	Alexa Fluor^®^ 488 Anti-Rabbit IgG	Donkey	ab150073	1:400	Abcam, Cambridge, UK

**Table 2:** Concordance between the immunofluorescence (protein) findings of the present study and transcript expression in two public STZ-induced diabetic rat renal-cortex datasets (early phase, GSE131221, ~3 weeks; mid phase, GSE7253, 6 weeks). Arrows denote the direction of change in diabetic relative to control; ≈unchanged denotes no significant difference. Fold changes and *p*-values (Mann–Whitney) are for the representative probe; full statistics are in Supplementary Tables S2 and S3.

Gene	IF Protein	Early mRNA (~3 wk)	Mid mRNA (6 wk)
*SDC1*	Early reactive increase, blunted later	↑ 1.53× (*p* = 0.003)	≈unchanged
*SDC2*	Sustained increase in diabetes (later)	≈unchanged	↑ 1.27× (*p* = 0.004)
*SDC4*	Preserved/no significant change	↓ 0.88× (*p* = 0.018)	↓ 0.87× (*p* = 0.002)
*NDST1*	Biphasic: up early, strongly down late	↓ trend (*p* = 0.07)	≈unchanged
*NDST2*	Modest increase early	↓ 0.91× (*p* = 0.005)	≈unchanged; age interaction

## Data Availability

The transcriptomic datasets analyzed in this study are publicly available in the NCBI Gene Expression Omnibus (https://www.ncbi.nlm.nih.gov/geo/; accessed on 12 June 2026) under accession numbers GSE7253 and GSE131221. The immunofluorescence data generated in this study are available from the corresponding author upon reasonable request.

## Supplementary Materials

The following supporting information can be downloaded at: https://www.mdpi.com/article/10.3390/cells15141277/s1, Figure S1: Transcript expression of the extended HS biosynthesis machinery (Ext1, Ext2, Glce, Hs2st1, Hs3st1, Hs6st1) and the neuronal syndecan SDC3 in STZ-induced diabetic rat kidney cortex at the early phase (GSE131221, ~3 weeks; Sham vs Diabetic) and mid phase (GSE7253, 6 weeks; Control vs Diabetic). SDC3 was available only at the mid phase; NDST3 and NDST4 were not represented on either platform. Boxplots show normalized log_2_ expression of the representative probe per gene; individual samples are overlaid. Significance (Mann–Whitney): * *p* < 0.05, ** *p* < 0.01, *** *p* < 0.001, ns = not significant; Table S1: Characteristics of the analyzed datasets; Table S2: GSE7253 (6 weeks)—five studied genes, diabetic vs non-diabetic, representative probe per gene. Values are mean ± SD of signal intensity; *p*-values Benjamini–Hochberg (BH)-corrected within the five-gene panel; Table S3: GSE131221 (~3 weeks)—five studied genes, diabetic (DKD) vs Sham, representative probe per gene. Values are mean ± SD of normalized log_2_ expression; *p*-values BH-corrected within the five-gene panel; Table S4: GSE7253 (6 weeks)—extended HS biosynthesis machinery and SDC3, diabetic vs non-diabetic. Values are mean ± SD of signal intensity; *p*-values BH-corrected within the extended panel; Table S5: GSE131221 (~3 weeks)—extended HS biosynthesis machinery, diabetic (DKD) vs sham. Values are mean ± SD of normalized log_2_ expression; *p*-values BH-corrected within the extended panel.

## Abbreviations

The following abbreviations are used in this manuscript:

DM	Diabetes mellitus
DN	Diabetic nephropathy
HSPGs	Heparan sulfate proteoglycans
SDC1	Syndecan-1
SDC2	Syndecan-2
SDC3	Syndecan-3
SDC4	Syndecan-4
HS	Heparan sulfate
ECM	Extracellular matrix
DM1	Diabetes mellitus type 1
DM2	Diabetes mellitus type 2
NDSTs	N-deacetylase/N-sulfotransferases
NDST1	N-deacetylase/N-sulfotransferase 1
NDST2	N-deacetylase/N-sulfotransferase 2
CTRL	Control
2 w	2 weeks
2 m	2 months
pH	Lat. potentia hydrogenii
PBS	Phosphate-buffered saline
DAPI	4′6-Diamidino-2-phenylindole
ANOVA	Two-way analysis of variance
pct	Proximal convoluted tubules
dct	Distal convoluted tubules
g	Glomerulus
mRNA	Messenger ribonucleic acid
TGF-β	Transforming growth factor-beta
IL-1β	Interleukin-1 beta
MMP	Matrix metalloproteinase
MMP-9	Matrix metalloproteinase-9
ERK1/2	Extracellular signal-regulated protein kinases 1 and 2
p38 MAPK	p38 Mitogen-activated protein kinase
STZ	Streptozotocin
IF	Immunofluorescence
Ext1	Exostosin glycosyltransferase 1
Ext2	Exostosin glycosyltransferase 2
Glce	D-glucuronyl C5-epimerase
NDST3	N-deacetylase/N-sulfotransferase 3
NDST4	N-deacetylase/N-sulfotransferase 4
Hs2st1	Heparan sulfate 2-O-sulfotransferase 1
Hs3st1	Heparan sulfate glucosamine 3-O-sulfotransferase 1
Hs6st1	Heparan sulfate 6-O-sulfotransferase 1
RNA	Ribonucleic acid
PCR	Polymerase chain reaction
