# Supplementary material for: Changes in Expression of Syndecans and Heparan Sulfate Biosynthesis Enzymes in Short-Term Streptozotocin-Induced Diabetic Rat Kidneys
Source: Cells. 2026 Jul 16;15(14):1277. doi: 10.3390/cells15141277 (PMC13407145; doi:10.3390/cells15141277)
Supplement: Supplementary file 1 [file cells-15-01277-s001.zip › cells-4416009-supplementary.pdf]

cells MDPI

Supplementary Materials

Table S1. Characteristics of the analysed datasets.

Feature	GSE7253 / GDS4038	GSE131221
Phase	Mid (6 weeks post-STZ)	Early (~3 weeks post-STZ)
Species / strain	Rat, Sprague-Dawley	Rat, Sprague-Dawley (Crl:CD)
Tissue	Renal cortex	Renal cortex
Model	STZ-induced diabetes	STZ-induced diabetes (65 mg/kg)
Platform	Affymetrix Rat230 2.0 (GPL1355)	Agilent SurePrint G3 Rat 8×60K
Value type	Signal intensity	Normalised log2
Groups compared	Diabetic (n=6) vs non-diabetic (n=6)	Diabetic/DKD (n=7) vs Sham (n=5)
Design note	2×2 (disease × age), n=3/cell	Enarodustat arm excluded

Table S2. GSE7253 (6 weeks) — five studied genes, diabetic vs non-diabetic, representative probe per gene. Values are mean ± SD of signal intensity; p-values Benjamini-Hochberg (BH)-corrected within the five-gene panel.

Gene	Probe	Diabetic	Non-diabetic	FC	MWU p	BH p
Sdc1	1367849_at	355.7 ± 89.7	345.6 ± 48.1	1.03	0.589	0.937
Sdc2	1382189_at	5313.2 ± 414.1	4185.3 ± 426.5	1.27	0.0043	0.011
Sdc4	1367721_at	10984.4 ± 618.4	12674.2 ± 577.8	0.87	0.0022	0.011
Ndst1	1387549_at	124.8 ± 15.3	145.7 ± 56.1	0.86	0.937	0.937
Ndst2	1374827_at	520.8 ± 37.1	529.7 ± 62.5	0.98	0.937	0.937

Two-way ANOVA disease main-effect p-values (log2): Sdc1 0.960, Sdc2 0.0033, Sdc4 0.0016, Ndst1 0.446, Ndst2 0.675; significant Ndst2 disease × age interaction p = 0.0013.

Table S3. GSE131221 (~3 weeks) — five studied genes, diabetic (DKD) vs Sham, representative probe per gene. Values are mean ± SD of normalised log2 expression; p-values BH-corrected within the five-gene panel.

Gene	Probe	DKD (diabetic)	Sham (control)	FC	MWU p	BH p
Sdc1	A_43_P14872	10.03 ± 0.29	9.41 ± 0.20	1.53	0.0025	0.013
Sdc2	A_43_P11701	10.20 ± 0.17	10.34 ± 0.09	0.91	0.202	0.202
Sdc4	A_42_P687186	12.52 ± 0.12	12.70 ± 0.05	0.88	0.018	0.030
Ndst1	A_44_P791351	8.35 ± 0.18	8.56 ± 0.11	0.87	0.073	0.092
Ndst2	A_44_P161389	9.66 ± 0.04	9.80 ± 0.06	0.91	0.0051	0.013

Table S4. GSE7253 (6 weeks) — extended HS biosynthesis machinery and SDC3, diabetic vs non-diabetic. Values are mean ± SD of signal intensity; p-values BH-corrected within the extended panel.

Gene	Probe	Diabetic	Non-diabetic	FC	MWU p	BH p
Ext1	1373393_at	570.2 ± 59.3	661.6 ± 70.8	0.86	0.041	0.144

Ext2	1371611_at	933.9 ± 68.6	1041.9 ± 95.6	0.90	0.041	0.144
Glce	1390385_at	466.6 ± 36.0	461.0 ± 39.2	1.01	0.818	0.818
Hs2st1	1382101_at	912.9 ± 78.3	1031.6 ± 334.9	0.88	0.394	0.552
Hs3st1	1370834_at	209.9 ± 27.1	263.1 ± 47.6	0.80	0.132	0.308
Hs6st1	1372467_at	617.4 ± 64.1	581.1 ± 140.2	1.06	0.818	0.818
Sdc3	1372180_at	106.2 ± 35.5	140.2 ± 17.9	0.76	0.240	0.420

Two-way ANOVA disease main-effect p-values (log2): Ext1 0.007, Ext2 0.068, Glce 0.813, Hs2st1 0.613, Hs3st1 0.060, Hs6st1 0.354, Sdc3 0.063. NDST3 and NDST4 were not represented on the platform.

Table S5. GSE131221 (~3 weeks) — extended HS biosynthesis machinery, diabetic (DKD) vs Sham. Values are mean ± SD of normalised log2 expression; p-values BH-corrected within the extended panel.

Gene	Probe	DKD (diabetic)	Sham (control)	FC	MWU p	BH p
Ext1	A_64_P158848	8.61 ± 0.09	8.53 ± 0.11	1.05	0.343	0.343
Ext2	A_44_P220277	7.44 ± 0.06	7.53 ± 0.06	0.94	0.048	0.088
Glce	A_42_P528106	7.71 ± 0.06	7.57 ± 0.09	1.10	0.003	0.015
Hs2st1	A_42_P775635	9.64 ± 0.16	9.46 ± 0.11	1.13	0.073	0.088
Hs3st1	A_44_P118666	6.55 ± 0.04	6.66 ± 0.06	0.93	0.010	0.030
Hs6st1	A_44_P145970	8.76 ± 0.11	8.60 ± 0.17	1.11	0.073	0.088

SDC3, NDST3 and NDST4 were not represented on the Agilent platform.

https://doi.org/10.3390/xxxxx

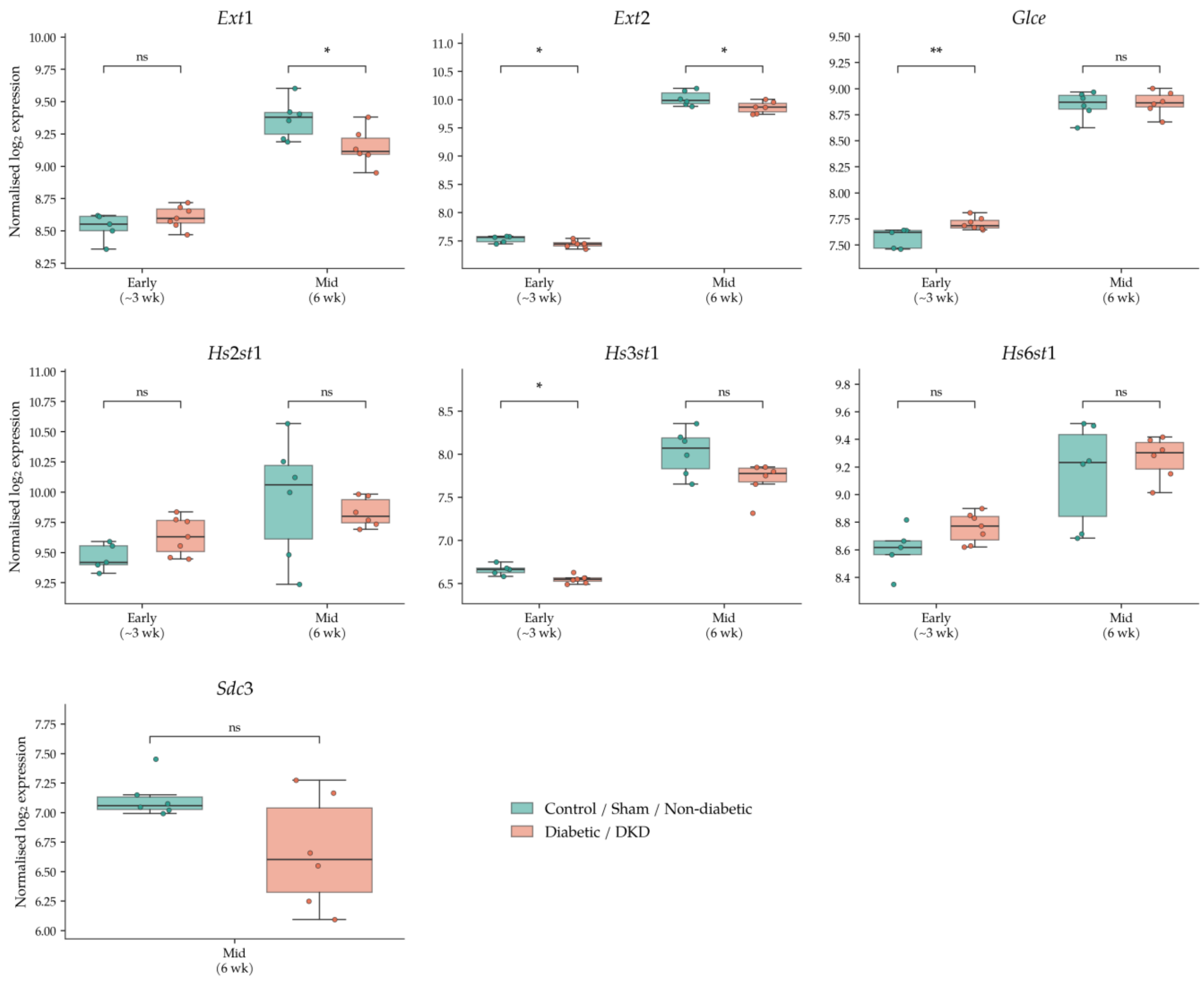

Figure S1. Transcript expression of the extended HS biosynthesis machinery (Ext1, Ext2, Glce, Hs2st1, Hs3st1, Hs6st1) and the neuronal syndecan Sdc3 in STZ-induced diabetic rat kidney cortex at the early phase (GSE131221, ~3 weeks; Sham vs Diabetic) and mid phase (GSE7253, 6 weeks; Control vs Diabetic). Sdc3 was available only at the mid phase; NDST3 and NDST4 were not represented on either platform. Boxplots show normalised log2 expression of the representative probe per gene; individual samples are overlaid. Significance (Mann-Whitney): *$p<0.05$, **$p<0.01$, ***$p<0.001$, ns = not significant.
